# Enteral formula compared to Nissen-Fundoplication: Data from a retrospective analysis on tolerance, utility, applicability, and safeness in children with neurological diseases

**DOI:** 10.3389/fnut.2023.1093218

**Published:** 2023-03-09

**Authors:** Chiara Maria Trovato, Teresa Capriati, Giulia Bolasco, Carla Brusco, Emma Santariga, Francesca Laureti, Carmen Campana, Valentina Papa, Bianca Mazzoli, Silvia Corrado, Renato Tambucci, Giuseppe Maggiore, Antonella Diamanti

**Affiliations:** ^1^Gastroenterology and Nutritional Rehabilitation Unit, Istituto di Ricovero e Cura a Carattere Scientifico (I.R.C.C.S.), Bambino Gesù Children's Hospital, Rome, Italy; ^2^Administrative Management, Istituto di Ricovero e Cura a Carattere Scientifico, Bambino Gesù Children's Hospital, Rome, Italy

**Keywords:** Enteral Nutrition, step-up strategy, Nissen Fundoplication, cost-analysis, neurological disorders

## Abstract

**Objectives and study:**

Approximately 46–90% of children with neurological disorders (NDs) suffer from gastrointestinal diseases, such as gastro-esophageal reflux disease (GERD), constipation, or malnutrition. Therefore, enteral feeding is often necessary to achieve nutritional requirements. The treatment of GERD could be based on pharmacological therapy, nutritional treatment (changing the type of formula), or surgical treatment (Nissen Fundoplication, NF). The aim of this study was to describe and compare resource consumption between NE based on different formulas and NF in patients with ND.

**Methods:**

We performed a retrospective analysis on all children with neurological damage (age: 29 days−17 years) treated from January 2009 to January 2019 due to nutritional problems and food and/or gastrointestinal intolerances. For all patients, demographic and anthropometric characteristics, symptoms, type of nutrition (formula and enteral access), and number and type of outpatient or emergency room visits were collected. Patients with <24 months of age at the closing of the database and with <24 months of follow-up were excluded.

**Results:**

Out of 376 children, 309 children (M: 158; median age: 4 IQR 1–10) were enrolled, among which, 65 patients (*NF group* M: 33; median age: 5.3 IQR 1.8–10.7) underwent NF. Vomit, GERD, and dysphagia were more represented in the NF group (*p* < 0.05). Our analysis shows that the NF group seems to present a lower number of hospitalization and a lower number of visits for non-GI disorders, but a higher number of visits for GI disorders compared to non-NF. In the NF group, a higher prevalence of the use of amino-acid-based formula and free diet is observed, with a trend for the lower prevalence of casein-based or whey+casein-based formula (Fisher test *p* = 0.072). The median cost of a patient enrolled in the database is € 19,515 ± 540 ($ 20,742.32 ± 573.96) per year, with no significant difference between the two groups. Regarding formula, at baseline, 76 children consumed a free diet, 24 a casein-based formula, 139 a whey+casein-based formula, 46 a whey-based formula, and 24 an amino-acid-based formula.

**Conclusions:**

In conclusion, compared to EN, NF may not improve the clinical aspect and related costs in children with NDs. Considering the psychological and QoL burden for patients, in a “step-up” strategy, EN could be proposed as an efficient alternative to NF.

## Introduction

Up to 90% of children with neurological disorders (NDs; both progressive, such as neurodegenerative disorders or mitochondrial disease, and non-progressive, such as cerebral palsy) can suffer from gastrointestinal diseases, such as gastro-esophageal reflux disease (GERD) and constipation, and malnutrition (1), associated with growth failure, micronutrient deficiencies, osteopenia, and other nutritional comorbidities (2). In these patients, several factors contribute to the onset of malnutrition, including inadequate dietary intake, occurring as a consequence of oral motor dysfunction due to the central nervous system or neuromuscular disorders. Moreover, the progression of oral motor dysfunction, which causes impairment of mastication and swallowing, can be due to weakness or impairment of oral function, tongue motor function, or chin motor function (3).

Gastrointestinal disorders can be related to the severity of the underlying neurological disability and the use of drugs, including antiepileptic agents (3), that could worsen gastrointestinal symptoms. Among gastrointestinal disorders, GERD is a well-described phenomenon (4, 5) but also the most frequent, affecting up to 70% of children with ND; it can cause aspiration, pneumonia, and death. Concerning GERD, pathophysiological mechanisms are multifactorial: the underlying neurological damage may cause delayed gastric emptying and esophageal dysmotility, while scoliosis, seizures, spasticity of the abdominal musculature, or constipation can all cause increased abdominal pressure. Due to their frequent profound physical disabilities, many children spend long periods in the supine position, thus minimizing the effect of gravity to aid esophageal clearance (6–8).

Several therapeutic options are available to improve the clinical conditions of these patients, and a trial with proton pump inhibitors (PPIs) can be considered as recommended first-line treatment (9). On the other hand, the routine use of prokinetic agents is not recommended, but their use may be considered in patients with uncontrolled GERD (9). Additionally, non-pharmacological treatments can also be considered, such as the inclusion of nutritional adaptation using enteral formulas containing whey proteins and surgical treatments (Fundoplication according to Nissen, NF) (9). On the other hand, NF, especially in patients with ND could be burdened by complications (10). Literature data report a prevalence of 1% of death (11) and 6% of major complications such as bleeding, sepsis, intestinal occlusion, esophageal perforation, and dumping syndrome (12–15) following NF in children with ND. Furthermore, neurological impairment has been suggested as a factor associated with mortality in children undergoing NF (15).

In children with ND who are fed by Enteral Nutrition (EN), modulating the type of formula could be considered an effective treatment option. By tube feeding, percutaneous endoscopic gastrostomy (PEG), or percutaneous endoscopic jejunostomy (PEJ), a wide variety of feeds are available, providing 1–1.5 kcal/ml (16), with or without fiber. Whey-based formulas have been shown to promote faster gastric emptying compared to casein-based formulas (17). The use of peptide-based, 100% whey protein formulas is associated with improved feeding tolerance, increased consistency in meeting nutritional needs, and a reduction in gastrointestinal issues like vomiting and aspiration of feeds (18).

This study aimed to describe and compare resource consumption in patients with ND treated with EN and with NF.

## Methods

### Patients

This study was designed as a monocentric, retrospective observational (*cohort*) study.

We retrospectively enrolled, between January 2009 and January 2019, all patients with ND (including progressive and non-progressive NDs) at the beginning of the Home Enteral Nutrition (HEN) program, performed through a nasogastric tube (NG) or gastrostomy (PEG) or gastro-jejunostomy (PEJ) and followed for at least 2 years after beginning HEN.

For all patients, demographic and anthropometric characteristics, gastrointestinal symptoms at enrolment, type of nutrition (formula and enteral access), and number and type of outpatient visits or emergency room access during the follow-up were collected.

Patients younger than 24 months or with follow-up shorter than 12 months were excluded.

The present study was approved by the Ethical Committee of Pediatric Hospital “Bambino Gesù” (2322_OPBG_2021) and submitted to Clinical Trial Gov (NCT05068089).

For clinical assessment, the following criteria were considered:

- Malnutrition has been diagnosed based on body mass index (BMI) *z*-score and weight-for-height/length for age *z*-score, or length/height-for-age or mid–upper arm circumference according to the WHO and CDC growth charts (2, 8–10) and the American Society for Parenteral and Enteral Nutrition (A.S.P.E.N.) consensus (19). Malnutrition was classified, based on BMI *z*- score and age *z*-score, as mild (between −1 and −1.9), moderate (between −2 and −2.9), and severe (>-3).
- Constipation was diagnosed based on clinical history (<3 stools per week) with or without suggestive imaging (abdominal radiograph).
- Dysphagia has been established based on multidisciplinary clinical evaluation (altered sucking/swallowing, cough during meals, and/or mealtimes more than 4 h/day) and/or on the videofluoroscopy findings.
- GERD has been diagnosed based on either partial or total remission of vomiting, regurgitation, discomfort, unexplained pain, retching, and bloating after proton pump inhibitor (PPI) treatment beginning with or without abnormal findings of esophago-gastro-duodenoscopy, gastric emptying-gastroesophageal reflux scintigraphy, upper gastrointestinal series, and/or 24-h esophageal pH-metry/pH-impedance study.
- All children undergoing NF after beginning HEN performed esophago-gastro-duodenoscopy with 24-h esophageal pH-metry/pH-impedance.

### Statistical analysis

Dependence between categorical data was measured using the chi-square test. The dependence of continuous variables with respect to categorical co-variates was evaluated using the *F*-test. Assessment of normal distribution was performed using the Shapiro–Wilk test for normality.

The categorical variables were represented in terms of absolute and relative frequencies (percentages), and the counts (number of outpatient visits, emergency department, and hospitalizations) were reported as annual rates [number of events over total person-years (***PY***) accumulated over the course of the study], while continuous variables were synthesized with median and interquartile range (IQR). For costs, it was decided to report the average cost per patient-year as the most frequently used statistical measure in the pharmacoeconomic field.

### Cost analysis

The cost analysis was based on the Italian National Health Service costs. Resource consumption was referred to as the annual rate of events (visits, hospitalization, and emergency room visits).

A unit cost corresponding to the current rate has been assigned to each resource consumed. The cost of each hospitalization was according to the specific “Diagnosis-Related Groups;” however, an average value of €6,000 ($6,377.35) was assigned to each admission for NF and €3,000 ($3,188.67) if the specific “Diagnosis-Related Group” was not available. The cost of each outpatient visit was evaluated at the rate of €20.66 ($21.96) [Decree 10/2012 GU SG 28/1/2013], while the cost for emergency room visits was estimated at €285 ($302.92) according to the rules of the Lazio region, where our institution is located. The Lazio region does not declare cost differences between polymeric formulas and semi-elementary formulas; so the cost for each HEN day was estimated at €28 ($29.76) regardless of the type of nutrition [Salis Document SINPE].

## Results

### Study population

Among 376 children, 309 children [M: 158; median age: 4 (IQR 1–10)] were enrolled, with a median follow-up of 5.47 years [IQR 3.73–7.99].

Sixty-seven patients were excluded due to the following: 7 were >18 years, 27 were younger than 24 months at the end of follow-up, and 52 were followed for <12 months.

Among 309 enrolled patients, 65 patients [***NF group*** M: 33; median age: 5.3 (IQR 1.8–10.7)] underwent NF; among them, 33 had undergone NF before starting HEN (median 8.7 months with IQR 0.3–8.8) and 32 after (median 28.5 with IQR 5.2–45.8), with a median follow-up of 7.5 years (IQR 5–8.3).

The cohort included 182 children (58.9%) with progressive ND, and 29 of them underwent NF.

Out of 244 children, in the **non-NF group** [M:125, median age 4.14 (IQR 1.14–9.88)], the median follow-up was 5.2 years (IQR 3.4–7.7).

With regards to NF complications, in our cohort, one patient died after NF for respiratory failure, and three patients underwent a second NF during the follow-up.

Table 1 shows the clinical characteristics of the study population.

**Table 1:** Clinical characteristics of the study population; all data were collected at enrollment.

	**Total (*N* = 309)**	**No NF group (*N* = 244)**	**NF group (*N* = 65)**	***p*-value**
**Baseline features**				
Age (median and IQR)	4.48 (1.26–9.88)	4.14 (1.14–9.88)	5.14 (1.8–9.88)	0.5173
Male	158 (51.13%)	125 (51.23%)	33 (50.77%)	NS
**Cause of neurological impairment** ^a^				0.924
Cerebral palsy				
Tetraparesis	282 (92%)	223 (92%)	59 (91%)	
Emiparesis	10 (3%)	8 (3%)	2 (3%)	
No limited mobility	16 (5%)	12 (5%)	4 (6.15%)	
Neurological progressive disease	182 (59%)	153 (63%)	29 (45%)	**0.011**
**Malnutrition**^b^ **(%)** **GI symptoms**^c^	201 (65%)	149 (61%)	44 (67%)	NS
Constipation	122 (40%)	95 (40%)	27 (42%)	NS
GERD	220 (71%)	159 (65%)	65 (100%)	**< 0.001**
Dysphagia	261 (86%)	199 (84%)	62 (95%)	**0.014**
**Comorbility** ^d^				
Tracheostomy	57 (19%)	49 (21%)	8 (12%)	0.154
Lung failure	59 (19%)	53 (22%)	6 (9%)	**0.021**

a Missing data for 55 patients in the non-NF group, nine in the NF group.

b Missing data for four patients in the non-NF group, one in the NF group.

c Missing data for five patients in the non-NF group.

d Missing data for six patients in the non-NF group.

GERD and dysphagia were significantly more frequent in the NF group (*p* < 0.05).

**Figure 1:**
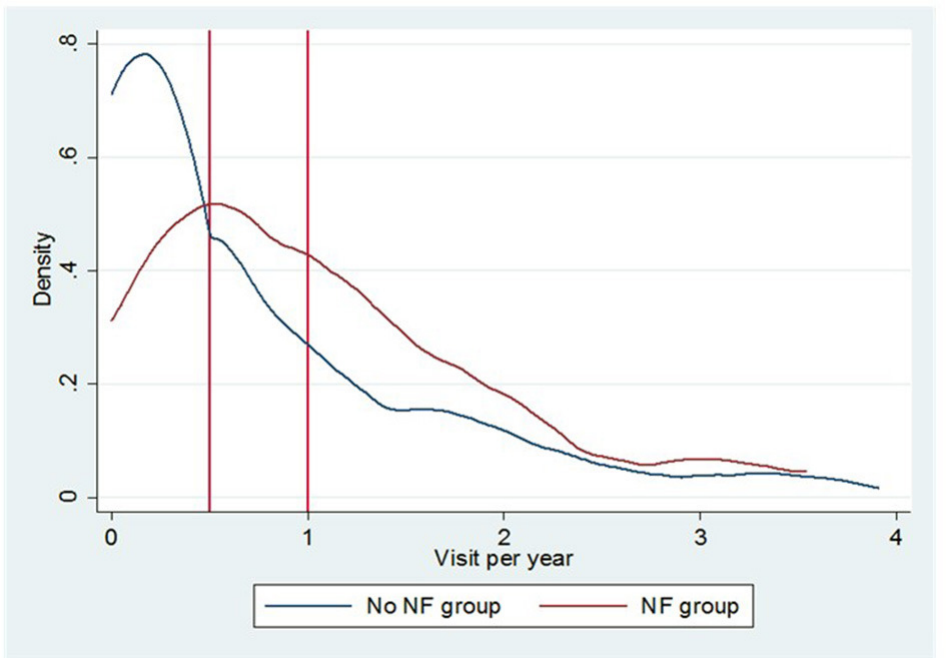
The Epanechnikov kernel density of annual outpatient visits for GI symptoms in the NF group and the non-NF-group.

Our analysis shows that the NF group, compared with the group treated with HEN alone, had a lower hospitalization rate and a lower rate of visits for non-GI disorders, but a higher rate of visits for GI disorders (Figure 1).

**Figure 2:**
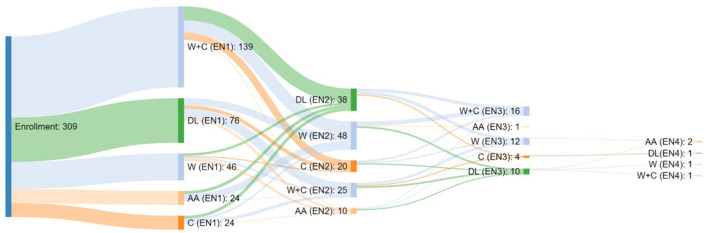
Type of Enteral Nutrition taken by the patients enrolled in the study and relative switch.

Concerning enteral formula, at baseline, 76 children were fed with homemade blenderized feeds, 163 with polymeric formula (24 casein, 139 whey+casein), 46 with hydrolyzed formula, and 24 with amino-acid (AA) based formula. During the study period, 54.4% of patients did not change EN, 32% changed EN type once, 12% changed EN type two times and 2% changed EN type three times (Figure 2).

At enrolment, in the NF group, we did not find significant differences in the use of amino-acid-based formula and homemade blenderized feeds when compared with casein and whey+casein-based formula.

All patients in the NF group continued to be fed by EN after surgery.

Regarding access for EN, at baseline, 12 (3.8%) patients were fed by NGT, 278 (90%) by PEG, and 19 (6.2) by PEJ. During the study period, 15 children out of 278 fed by PEG changed enteral access, switching to PEJ. In particular, in the **NF group**, five out of 52 children fed by PEG at baseline switched to PEJ) and six were fed by PEJ at baseline.

**Figure 3:**
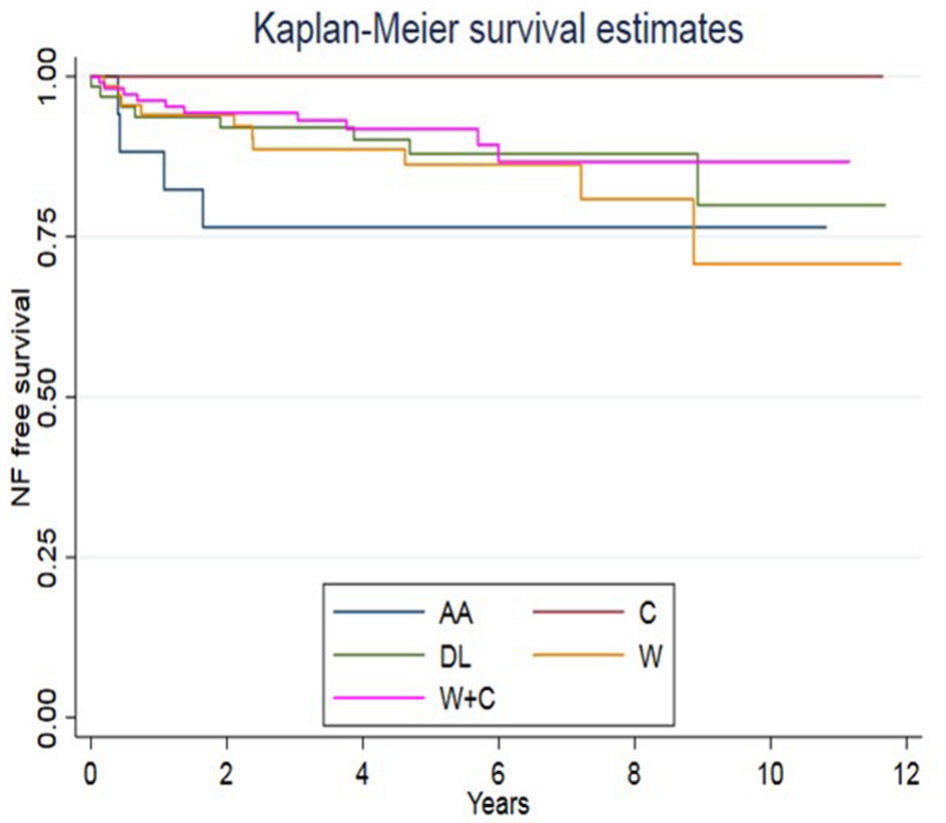
Types of formula and switch assumed in our cohort. At baseline, 76 children were fed with a free diet (homemade blenderized feeds), 24 casein, 139 whey+casein, 46 whey, and 24 amino-acid-based formulas.

Figure 3 shows the Kaplan–Meier curve on surgery-free survival as a function of the “leading Enteral Nutrition type” (the one followed for the longest time). This curve shows that the AA-fed group underwent NF earlier than other feeding groups; the log-rank test, nevertheless, did not display significant differences between formulas (*p* = 0.0961).

### Cost analysis

The cost analysis was based on the annual rate [number of events over total person-years (***PY***) accumulated over the course of the study].

As shown in Figure 1, the NF group had a higher incidence of outpatient visits for GI symptoms [1.15 vs. 0.73 visits for PY, incident rate ratio (IRR) = 1.12; 95% CI 0.97–1.30] and a lower incidence of visits for other causes (1.74 vs. 2.04 visits for PY, IRR = 0.85; 95% CI 0.76–0.95). Overall, the emergency room visits were similar between the groups. There is also a lower incidence of hospital admissions in the NF group (1.21 vs. 1.48 events for PY, IRR = 0.89; 95% CI 0.78–1.01).

The annual cost for each patient enrolled was €19,515 ± 540 ($20,742.32 ± 573.96) [€19,508 ± 655 ($20,734.88 ± 696.19) for EN alone and €17,772 ± 910 ($18,889.70 ± 967.23) for the NF group]. These results show that the median cost of each patient in the NF group was lower than in the non-NF group [–€1,736 ($1,850); 95% CI - €4,235 ($4,501.34) to + €763 ($810.99)], but these data do not reach a significance.

This difference appears less marked [–€748 ($795.04); 95% CI - €4,487 ($4,769.19) to 2,992 ($3,180.17)] when corrected for main confounders (age, sex, severity nutritional status, comorbidities, GI symptoms at enrolment, progressive neurological disease, and proximity to the center), as shown in Table 2.

**Table 2:** Cost analysis based on annual rates in study cohort.

**Annual cost per patient**	**Total (*N* = 309)**	**No NF (*N* = 244)**	**NF (*N* = 65)**	**Delta**	**Delta adjusted^a^**
Enteral Nutrition (€)	9.794 ± 92.3	9.830 ± 96.0	9.660 ± 251.7	−170.00 (−611 to 271)	−81.92 (−693 to 529)
Outpatient visits (€)	59.20 ± 5.2	59.26 ± 6.3	58.95 ± 5.9	−0.32 (−25 to 24)	−0.43 (−33 to 32)
Emergency room (€)	170.93 ± 18.6	171.21 ± 21.2	169.87 ± 38.8	−1.34 (−90 to 88)	−1.02 (−117 to 115)
Hospital admissions (€)	9.119 ± 530	9.448 ± 631	7.883 ± 844	−1.564 (−3.836 to 708)	−1.574 (−6.314 to 3.165)
Total (€)	19.143 ± 552	19.508 ± 655	17.772 ± 910	−1.736 (−4.235 to 763)	−748 (−4.487 to 2.992)

a Adjusted for age, sex, severity (tetraparesis, hemi, or other paresis), nutritional status, comorbidities, GI symptoms at enrollment, proximity to the center, and progressive neurological disease.

Furthermore, a cost analysis was done on the 65 patients of the NF group, among which, 32 underwent NF after beginning HEN. Therefore, we compared resource consumption between patients who underwent NF before and after beginning HEN. We found that the rate of hospitalization was slightly reduced in a patient undergoing NF after beginning HEN (1.15 vs. 1.48 events per PY, IRR = 0.83; 95% CI 0.65–1.08).

After adjusting for main confounders, nevertheless, the differences in resource consumption between the non-NF group and the NF group before beginning HEN and the non-NF group and the overall NF group were not statistically significant (delta adjusted –€ 751; 95% CI −1.663 to 3.165 and –€ 748; 95% CI −4.487 to 2.992, respectively; see Table 2 for details).

## Discussion

Gastrointestinal symptoms and malnutrition are frequent co-morbidities in ND children and are very challenging in their clinical management. GERD, constipation, and dysphagia decrease the QoL of children and families (9). This study's population represents a snapshot of children with neurological impairment, proving that some symptoms (such as vomiting, GERD, and dysphagia) are more frequent in children needing NF. The presence and persistence of these symptoms, indeed, can promote the progression of the disease, therefore making NF surgery mandatory.

The most original finding of the present study is that we did not find differences in the estimated healthcare costs between the NF- and non-NF groups. Interestingly, our observations may suggest that EN can be an effective therapeutic option to delay surgery and its associated complications without increasing healthcare costs. Furthermore, the number of emergency room visits (for gastrointestinal complications or other) was similar between the two groups.

Patients in the NF group had a higher incidence of outpatient visits for digestive complications and a lower incidence of visits for other causes. These findings suggest that NF cannot improve gastrointestinal symptoms, in agreement with previous studies (19, 20). NF is associated with morbidity and mortality related to relapse of GERD in 12–30% of the patients and, in particular, it seems to fail in 40% of the cases (20, 21). Therefore, NF with the aim of preventing GERD leads to irreversible alterations of the gastro-esophageal anatomy and function (22). Moreover, following NF, gas bloat syndrome, impaired gastric accommodation, gastric hypersensitivity, rapid gastric emptying (or “dumping syndrome”), retching, or dysphagia (10, 23) can occur.

Neurologically impaired children are at higher risk of death following NF and of failure of the surgery (15) and this should suggest the need for an alternative approach to NF in patients with ND and GERD. Interestingly, recently published data suggests that if NF fails and patients are free from GERD symptoms on jejunal feeding, further anti-reflux surgical intervention should be avoided (24). Another recent study suggests that EN alone, without combined surgery, could improve digestive symptoms (25). Furthermore, the combination of PEG with NF has been considered neither mandatory nor efficacious in preventing GERD in infants with NDs (26).

Conflicting results have been reported regarding the benefits of NF on the quality of life (QoL) of patients with ND. Some studies showed improved QoL (27, 28), but a more recent paper (29) demonstrated that QoL improved 3 months after NF but it dropped 5 years after surgery.

Unlike the above-reported study, in the present research, we mostly evaluated healthcare costs for EN and NF, and we showed that EN alone does not increase healthcare costs, because the rate of complications needing hospitalization, emergency room visits, or outpatient visits is similar in both the non-NF and NF groups.

Therefore, EN can be considered the primary therapeutic option to delay surgery in ND children with GERD, and the switch to a different enteral formula could be appropriate to improve tolerance and reduce digestive symptoms (30, 31).

**Figure 4:**
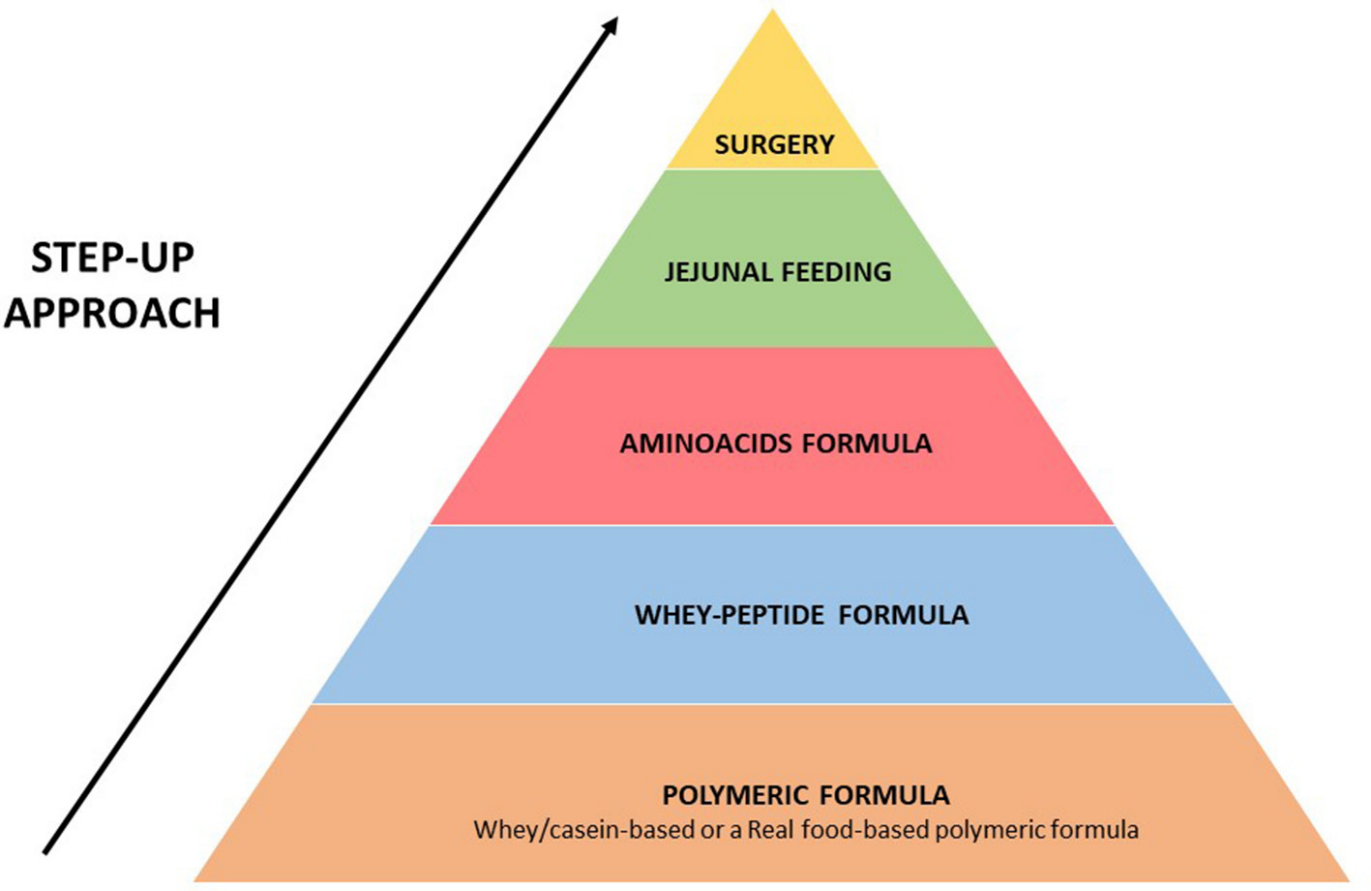
Proposed “step-up strategy” for the management of patients with persisting symptoms of GERD and fed with enteral formula.

We hypothesize that a “step-up” strategy (Figure 4), like in inflammatory bowel disease, could be proposed for patients with persisting symptoms of GERD who are fed with polymeric formula (whey/casein-based polymeric formula or a real food-based polymeric formula).

Some evidence showed that whey-based formulas improve gastric emptying if compared with casein-based formulas (17, 32). Furthermore, increased absorption of hydrolyzed over intact proteins may be observed in certain patient populations (33). Peptide-based 100% whey protein formulas are associated with improved feeding tolerance and reduced gastrointestinal symptoms, such as vomiting and aspiration (31).

Furthermore, a growing body of literature reports that “real food-containing formulas” improve tolerance and feeding outcomes, mimicking “real nutrition,” stimulating gut hormone production, and improving motility (34–37).

Together with the modulation of formula feeding, a further important step that can improve tolerance to EN in patients with severe GERD is the switching from gastric to jejunal feeding (given continuously) (18, 24, 38) (see Figure 4 for details).

To the best of our knowledge, the present study is the first to compare the true costs of nutrition and surgery in the management of patients with ND. Up until now, cost-analysis studies compared different surgical approaches (robotic vs. laparoscopic vs. open strategy) (39–41) or surgery and medical treatment (e.g., PPI treatment) (42–44).

Some limitations are present in this study given that it analyzes the costs deriving from only the Italian Health System and therefore represents a snapshot of what is happening in the country. The Italian Health System differs from other types of health care and, therefore, our cost analysis could be different in other European and non-European countries. Furthermore, we acknowledge limitations on sample size and the lack of effective control measures for individual heterogeneity, especially related to hospitalizations.

## Conclusion

In conclusion, we found that healthcare costs and the rates of hospitalization and emergency room and outpatient visits seem to be similar in patients who receive only EN compared to those receiving both surgery and EN. Furthermore, NF does not seem to allow for the suspension of HEN, so costs related to nutritional treatments remain unchanged. Therefore, EN could be considered the primary approach for ND patients with GERD, while NF should be seen as the last option after changing the type, site, and method of EN administration. Furthermore, perspectives on health economics implications are under assessment.

## Data availability statement

The raw data supporting the conclusions of this article will be made available by the authors, without undue reservation.

## Ethics statement

The studies involving human participants were reviewed and approved by 2322_OPBG_2021. Written informed consent to participate in this study was provided by the participants' legal guardian/next of kin.

## Author contributions

AD and CMT conceptualized and designed the study, coordinated and supervised data collection, drafted the initial manuscript, and reviewed and revised the manuscript. TC, CB, and GB designed the data collection instruments. ES, FL, CC, VP, BM, and SC collected data and carried out the initial analyses. CMT and GB coordinated and supervised data collection and designed and revised the statistics. RT, GM, and AD supervised the correct definition of outcomes and the subgroups critically reviewed the manuscript for important intellectual content. All authors approved the final manuscript as submitted and agree to be accountable for all aspects of the work.
